# Protective Effect of Magnesium Isoglycyrrhizinate on Ethanol-Induced Testicular Injuries in Mice

**DOI:** 10.1016/S1674-8301(10)60024-3

**Published:** 2010-03

**Authors:** Yuanqiao He, Fuqing Zeng, Qing Liu, Wen Ju, Houju Fu, Hua Hao, Lulu Li, Yifeng Xie

**Affiliations:** a Department of Urology; b Department of Obstetrics and Gynecology; d Department of Integrated Chinese Medicine and Western Medicine, Union Hospital, Tongji Medical College, Huazhong University of Science and Technology, Wuhan 430022, Hubei Province, China; c Department of Pathophysiology, Tongji Medical College,Huazhong University of Science and Technology, Wuhan 430030, Hubei Province, China

**Keywords:** ethanol, oxidative damage, testicular injury, magnesium isoglycyrrhizinate

## Abstract

**Objective:**

Ethanol treatment induces an increase in oxidative stress. As licorice compounds are potent antioxidants, our aim was to examine whether magnesium isoglycyrrhizinate attenuated lipid peroxidation, the major end-point of oxidative damage resulting from ethanol administration.

**Methods:**

Four groups(18 animals in each group) of male Kunming mice were used. The first group served as control and received 0.4 ml normal saline daily for 18 days orally. The second group of mice was given 56% ethanol at 16 ml/kg body weight per day for 18 days orally. The third group was given the same dose of ethanol and administrated magnesium isoglycyrrhizinate (15 mg/kg.d, i.p.) for 18 days. The fourth group was given the same dose of ethanol and administrated with magnesium isoglycyrrhizinate (45 mg/kg.d, i.p.) for 18 days. Twenty four hours after 9 days or 18 days of treatment the mice were sacrificed using 10% chloral hydrate. Sperm counts and motility in the epididymis were assessed. The lipid peroxidation and antioxidants of testicular mitochondria were also determined. The pathological changes of testicle tissue of the mice were observed by light microscopy.

**Results:**

Magnesium isoglycyrrhizinate effectively prevented the ethanol-induced seminiferous epithelium disorganization and degeneration of Sertoli cells and germ cells. Sperm counts and motility of the magnesium isoglycyrrhizinate treated groups were higher than those of the alcohol treated group, but were lower than those of the control group. The drug exhibited an ability to counteract ethanol induced oxidative challenge as it effectively reduced testicular malondialdehyde (MDA) and increased the activities of superoxide dismutase and glutathione peroxidase.

**Conclusion:**

Magnesium isoglycyrrhizinate is able to inhibit the ethanol-induced lipid peroxidation and has a protective effect against testicular oxidative injury.

## INTRODUCTION

Ethanol is one of the most widely abused drugs in the world. Chronic alcohol abuse leads to both liver injury and impaired sperm production in humans and animal models[1]. Some studies indicated that the testis may be even more sensitive to the effects of ethanol than the liver[2],[3]. Alcoholics were often found to have fertility abnormalities and impotence[4]. Testicular atrophy of these patients demonstrated a marked reduction in seminiferous tubular diameter accompanied by a loss of sperm cells[5]. Alcohol intake affects the activity of the hypothalamo-pituitary-gonadal (HPG) axis[6], and chronic ethanol ingestion results in significant alterations in sex hormone levels and sex hormone-responsive phenotype[4].

The mechanisms of ethanol induced testicular injury was previously studied. One proposed mechanism is that ethanol metabolism produces an oxidative stress within the testes[7], and another is that defective steroid hormone metabolism is secondary to altered liver metabolism[8]. In tissues, an imbalance between ROS production and antioxidant defense has been shown to result in cellular damage via lipid peroxidation, protein oxidation and DNA degradation[9]. Mitochondria-enriched extracts obtained from the testes of alcohol treated rats showed a significant increase in malondialdehyde (MDA) formation accompanied by a significant decrease in superoxide dismutase (SOD) and glutathione peroxidase[10].

Licorice root is a traditional herbal remedy that has been used to reduce liver injury in a number of clinical disorders. Magnesium isoglycyrrhizinate (MgIG) is a magnesium salt of 18-α glycyrrhizic acid stereoisomer, which is extracted from the roots of the plant *Glycyrrhiza glabra* (licorice). The structure of MgIG is tetrahydrate magnesium 18α, 20β-hydroxy-11-oxo-norolean-12-en-3β-yl-2-O-β-D-glucopyranurosyl-α-D-glucopyranosiduronate[11]. Increasing evidence supports the hypothesis that MgIG protects against several models of oxidant-mediated toxicity, including exposure to carbon tetrachloride and D-galactosamine. MgIG generally exhibits greater hepatic protection and anti-inflammatory activity than glycyrrhizin and β-glycyrrhizic acid[12],[13].

MgIG has an obvious therapeutic effect on chronic hepatitis and cirrhosis, and is a safe, well-tolerated drug[11],[14]. About three fourths of the male patients with advanced alcoholic cirrhosis have been reported to have testicular atrophy[15]. In view of the possible role of the liver in ethanol induced testicular injury the present study was designed to investigate the effect of MgIG on ethanol induced testicular injury in Kunming mice.

## MATERIALS AND METHODS

### Materials

Injectable magnesium isoglycyrrhizinate (50 mg:10 ml ) was purchased from Chia-tai Tianqing Pharmaceutical Co., Ltd, China. 56%(v/v) Red Star Erguotou liquor was purchased from Beijing Red Star Co., Ltd., China. SOD, MDA and Glutathione Peroxidase (GSH-PX) Detection Kits were purchased from Nanjing Jiancheng Bioengineering Institute, China. HEPES buffered Tyrode's lactate and bovine serum albumin (BSA) were purchased from Sigma-Aldrich Chemical Co, USA. The in Situ Cell Death Detection Kit was purchased from Roche Ltd., USA.

### Animals

Adult male Kunming mice weighing 20-25 g, purchased from the Laboratory Animal Center of Tongji Medical College, Huazhong University of Science and Technology (China), were used in this study. The animals were housed in plastic cages in a well ventilated room, and all animals were fed the same diet for 1 week before and during the experiment. The animals were also maintained at a temperature of 22±2°C with a 12 h light/dark cycle. All mice had free access to a standard diet and tap water. The experiment was carried out with the approval of the local animal use committee.

### Experimental design

After a habituation period, the mice were divided into four groups of 18 animals each: The first group (normal control, NC) served as the normal control and received 0.4 ml normal saline by gavage for 18 days, using a 12 gauge stainless steel blunt tipped needle. The second group (model control, MC) was administered 56%(v/v) alcohol, 16 ml/kg.d by gavage for 18 days. The third group (MgIG low dose group, ML) was given the same dose of alcohol and administrated with MgIG (15 mg/kg.d, i.p.) for 18 days. The fourth group (MgIG high dose group, MH) was given the same dose of alcohol and administrated MgIG (45 mg/kg.d, i.p.) for 18 days. MgIG was given at 17:30 every day, 30 minutes before the administration of alcohol. After 9 days and 18 days treatment the mice were sacrificed using 10% Chloral Hydrate. The testes and epididymis were removed, the adhering tissues were removed, and the specimens rinsed with ice cold normal saline, trimmed and processed for biochemical analysis and histomorphology studies.

### Testis histopathologic evaluation

The testes were removed and placed in 4% paraformaldehyde for 6 hours and subsequently paraffin embedded. Sections of 4 µm were cut and stained by haematoxylin eosin using a standard procedure. The light microscope histologic evaluation was performed by a pathologist in a blind, randomly numbered fashion without knowledge of the animal treatment group.

Testicular injury and spermatogenesis were graded in each group on the basis of the Johnsen score[16]. Score 1: Elements of the seminiferous epithelium were lacking and tubular sclerosis was evident. Score 2: no germ cells, only Sertoli cells. Score 3: spermatogonia only. Diameter of the tubule was reduced. Score 4: no spermatids, few spermatocytes, and arrest of spermatogenesis at the primary spermatocyte stage. Score 5: no spermatids and many spermatocytes. Score 6: no late spermatids, few early spermatids, arrest of spermatogenesis at the spermatid stage, and disturbance of spermatid differentiation. Score 7: no late spermatids and many early spermatids. Score 8: few late spermatids. The tubule reflects the status of hypospermatogenesis. Score 9: Many late spermatids and disorganized tubular epithelium. Score 10: full spermatogenesis.

### In situ TUNEL staining and quantitation

Paraffin-embedded sections, 4 mm thick, were cut onto silane-coated glass slides and a TUNEL assay was performed using an In Situ Cell Death Detection Kit. Tissues were counterstained with hematoxylin, then examined and photographed using an Olympus microscope. For statistical analysis, the percentage of seminiferous tubules containing three or more apoptotic cells of the total number of seminiferous tubules were calculated.

### Sperm counts

All cauda epididymides were excised and then placed in 35 mm Petri dishes containing HEPES buffered Tyrode's lactate (TL-HEPES) solution with 3 mg/ml BSA. Each cauda epididymides was then cut into several pieces using fine scissors to allow sperm to swim out for 10-15 min at 37°C. From the sperm suspension, 20 µl aliquots were placed on the neubauer hemacytometer to count the number of sperm of the cauda epididymis tissue. Sperm motility characteristics were subsequently evaluated by using the computer-assisted sperm analysis (CASA) system.

### Lipid peroxidation studies

The lipid peroxidation product in the testes was determined by thiobarbituric acid reactive substances (TBARS), and expressed as the extent of MDA production[17]. Testes were homogenized in ice-cold 0.25 M Tris buffer (pH 7.4). To this homogenate, TCA-TBA-HCl [Trichloroacetic acid (TCA) 15% w/v, thiobarbituric acid (TBA) 0.375%, and hydrochloric acid (HCl) 0.25 N] were added and mixed thoroughly. The solution was heated in a water bath at 95°C for 40 min. After cooling, the flocculent precipitate was removed by centrifugation at 1,500 g for 10 min. Levels of TBARS were measured spectrophotometrically at 532 nm. Tissue protein was measured by the Bradford method using BSA as standard.

### Antioxidant enzyme assays

Tissue homogenates (25 w/v) were prepared in 50 mM Tris-HCl buffer (pH 7.4) using a motor driven teflon homogenizer. Homogenates were centrifuged for 30 minutes (4°C, 13,400 g) and the resultant supernatant fractions were used for various assays. Superoxide dismutase and glutathione peroxidase were determined following the manufacturer's protocols.

### Statistical analyses

All data are expressed as mean ±SD. Differences among groups were analyzed by one-way analysis of variance (ANOVA), and the post hoc Student-Newman-Keuls (SNK) method was used for multiple comparisons. The *P* value reported was two-sided and a value of *P* < 0.05 was considered statistically significant. All analyses were performed using the SPSS software (Version 12.0, SPSS Inc., USA).

## RESULTS

### Overall Animal Health

Seventy-two mice entered the study, approximately 20% of the animals died during the study. 6 MC group mice, 4 ML group mice and 3 MH group mice died, but no deaths were recorded in the normal control group.

### MgIG attenuated ethanol-induced pathological changes in the testis

**Fig. 1:**
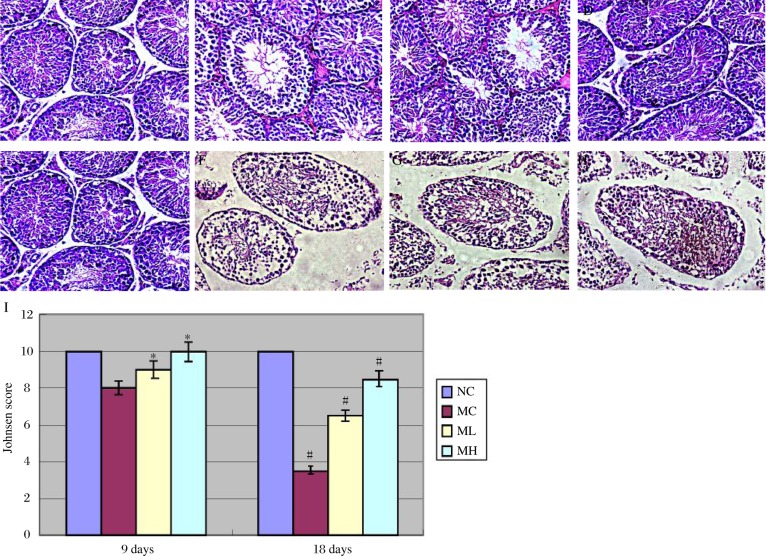
Pathological changes of testes (A-H,400×) and Johusen scores (I) after exposure to ethanol for 9 days (A-D) and 18 days (E-H). A and E: NC group. B and F: MC group. C and G: ML group. D and H: MH group. I: the Johnsen scores after 9 days and 18 days of ethanol treatment, *P < 0.05 (MC vs NC and MH vs MC, after 9 days of ethanol treatment; ^#^*P* < 0.01 (MC vs NC, MH vs MC, ML vs MC, after 18 days of ethanol treatment.

**Fig. 2:**
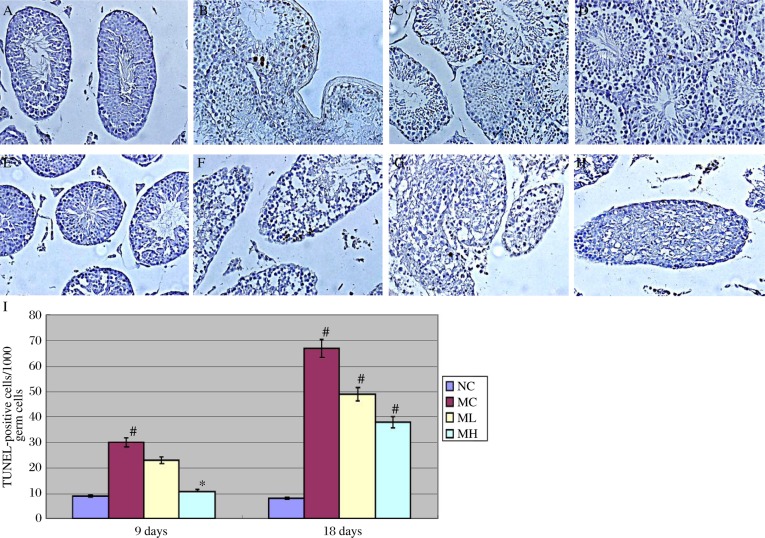
Representative photomicrographs of TUNEL-stained (Arrows) testis sections (A-H, 400×) and statistal anaylsis (I) after exposure to ethanol for 9 days (A-D) and 18 days (E-H). A and E: NC group. B and F: MC group. C and G: ML group. D and H: MH group. I: TUNEL-positive cells in mice testes following ethanol treatment, *P < 0.05 (MH vs MC, after 9 days of ethanol treatment ); ^#^*P* < 0.01, (MC vs NC, after 9 days of ethanol treatment ; MC vs NC, MH vs MC, ML vs MC, after 18 days of ethanol treatment).

Ethanol treatment induced severe injury of spermatogonia and spermatocytes in MC mice. MC mice showed a disorganized tubular epithelium and few late spermatids at the 9 day time point, and then spermatids, few spermatocytes, and arrest of spermatogenesis at the primary spermatocyte stage at the 18 day time point. The seminiferous epithelia of the NC mice at both time points showed undisturbed spermatogenesis and no obvious degenerative changes. After ethanol exposure, MH mice showed nearly normal testicular morphology, whereas ML mice displayed moderate injury of spermatogonia and spermatocytes. In some regions, expanded lumen and degenerative changes of epithelial component of the tubules occurred. ***Fig. 1 A-H*** show representative photomicrographs of testes from the four groups of mice after 9 days and 18 days of ethanol treatment.***Fig. 2 I*** summarizes the Johnsen scores after 9 days and 18 days of treatment.

### MgIG decreased ethanol-induced germ cell apoptosis

Apoptotic germ cells could be labeled using the TUNEL method. Within the testes of NC mice, only a few TUNEL-positive cells were observed. In MC and ML mice, ethanol treatment resulted in significantly increased numbers of TUNEL-positive cells per tubule compared to NC mice. Increases in TUNEL-positive cells were also observed 9 days and 18 days after ethanol treatment of MH mice in comparison to NC mice, although the increases did not reach significance at the 9 day time point. The numbers of apoptotic cells per tubule were increased in the ML mice testes following ethanol treatment, but the number of apoptotic cells was only half of that in the MC mice testes. (***Fig. 2A-2H***)

For statistical analysis, a scoring system based on the ratio of TUNEL-positive cells per 1,000 germ cells was used. TUNEL-stained multinucleated cells were counted as a single event. ***Fig. 2 I*** quantifies TUNEL-positive cells in four groups at 9 days and 18 days after ethanol treatment. MH mice testes displayed a significant reduction of apoptosis compared to MC mice after ethanol exposure, which suggested that MgIG decreased ethanol-induced germ cell apoptosis.

### MgIG prevented ethanol-induced decreases of sperm counts and motility

**Fig. 3:**
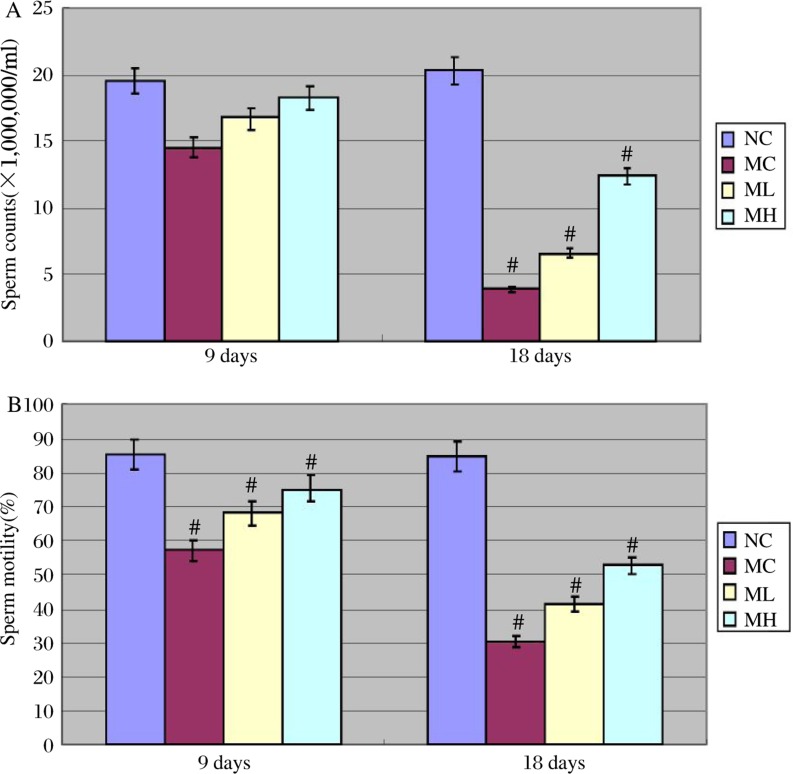
Sperm counts (A) and sperm motility (B) after 9 days and 18 days of ethanol treatment. ^#^*P* < 0.01 ( MC vs NC, MH vs MC, ML vs MC, after 9 days and 18 days of ethanol treatment).

Compared with NC mice, MC mice showed a sharp decrease of sperm counts and motility after ethanol treatment. ML and MH mice also showed a decrease of sperm counts and motility after ethanol treatment when compared to NC mice, but no statistical difference was observed between MH and NC mice at the 9 day time point. MH and ML mice showed greater sperm motility than MC mice. (***Fig. 3***)

### MgIG inhibited ethanol-induced oxidative stress in the testis

**Fig. 4:**
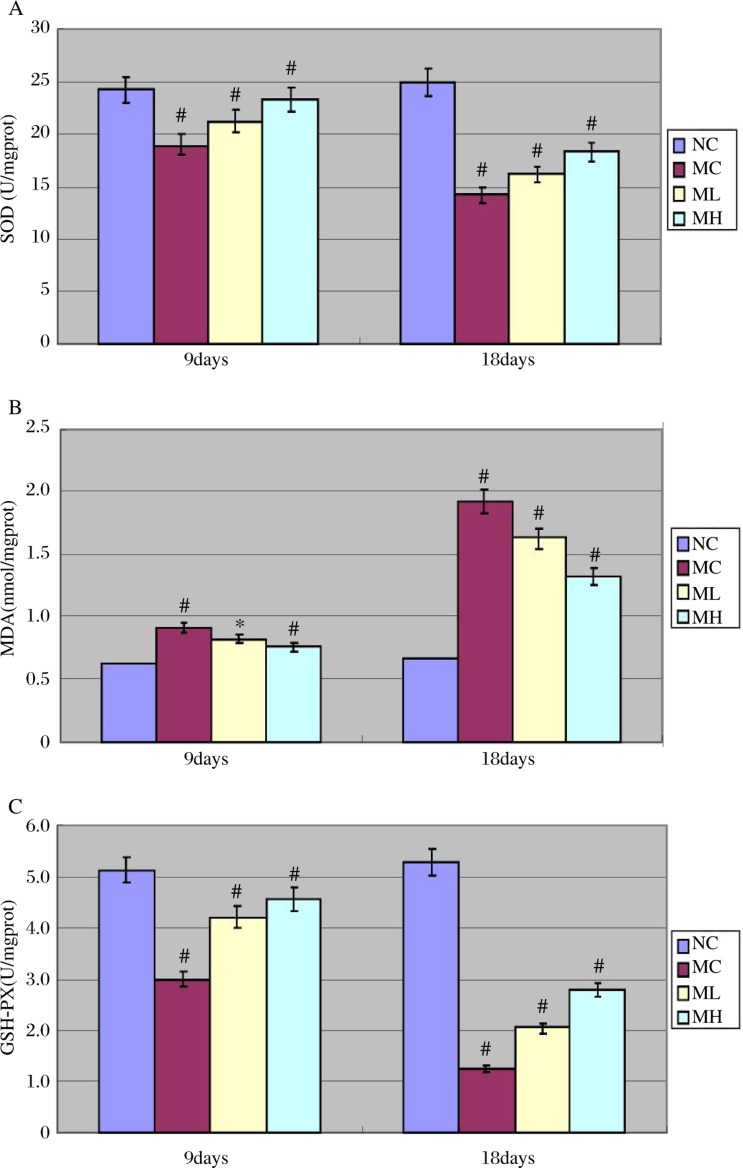
SOD activity (A), MDA(B) and GSH-PX (C) after 9 days and 18 days of ethanol treatment. A:SDD activity. ^#^*P* < 0.01 ( MC vs NC, MH vs MC, ML vs MC, after 9 days and 18 days of ethanol treatment). B: MDA. *P < 0.05 (ML vs MC, after 9 days of ethanol treatment ); ^#^*P* < 0.01 ( MC vs NC, MH vs MC, after 9 days of ehanol treatment). ^#^*P* < 0.01 ( MC vs NC, MH vs MC, ML vs MC, after 18 days of ethanol treatment). C: GSH-PX. ^#^*P* < 0.01 ( MC vs NC, MH vs MC, ML vs MC, after 9 days and 18 days of ethanol treatment).

MC mice demonstrated significantly greater lipid peroxidation (MDA) in the testes after ethanol treatment. The activities of SOD and GSH-Px were found to be significantly decreased in MC mice. MgIG exhibited an ability to counteract the ethanol-induced oxidative challenge as it effectively reduced testicular MDA and increased the activities of SOD and GSH-Px. (***Fig. 4***)

## DISCUSSION

Our data were consistent with previous work on ethanol-induced toxicity in testes. In our study, increased oxidative stress and germ cell apoptosis were observed after ethanol treatment. Furthermore, we observed severe injury in spermatocytes in some regions and degenerative changes of epithelial component of the tubules in MC mice testes after ethanol exposure.

Chronic ethanol abuse leads to testicular atrophy and male infertility in alcoholic men. It is well known that ethanol exposure disrupts the HPG axis, adversely affects the secretory function of Sertoli cells, and produces oxidative stress within the testes[10],[18]. The production of reactive oxygen species (ROS) is kept at physiologically low levels, and serves as a second messenger system in many different cell types. It was reported that excessive ROS production beyond critical levels overwhelmed the antioxidant defense strategies of spermatozoa in seminal plasma and resulted in increased oxidative stress[15]. Furthermore, increases in germ cell apoptosis were observed after testicular injury or exposure to various testicular toxicants in human and laboratory animals[19]. Some studies showed that apoptosis was an important mechanism by which ethanol caused tissue injury, and ethanol enhanced apoptosis of testicular germ cells[20].

The hepatoprotective effect of MgIG has been well documented and seems to be related to its antioxidant properties. MgIG treatment significantly ameliorated CCl4 -induced biochemical alterations and lipid peroxidation in the liver of mice or rats[12],[13],[21]. The protective effect of MgIG can be explained by the scavenging of free radicals before the damage to cellular macromolecules.

We are currently investigating the mode of action of MgIG as well as the putative involvement of detoxifying enzymes, such as superoxide dismutase and glutathione peroxidase, which are known to be implicated in severe testicular injury. Our data is the first report that MgIG, a pure magnesium salt of 18-α glycyrrhizic acid stereoisomer, inhibited ethanol-induced testicular injury in mice. The formation of MDA was significantly elevated, while SOD and GSH-PX activity decreased in testicular cells after exposure to ethanol. However, all these parameters exhibited recovered when the ethanol exposure was combined treatment with MgIG.

Our study demonstrated the adverse effect of ethanol on testicular sperm formation and motility and the protective effect of MgIG administration. In addition, MgIG also ameliorated the ethanol-induced germ cell apoptosis. MgIG may execute its role by modulating testicular free radical production and/or expression of some proteins, such as Bcl-2, p53, NF-κB[12],[13].

The time points of ethanol administration in this study, 9 days and 18 days, were selected to mimic the spermatogenic cycle of the mouse. The spermatogenic cycle length of the mouse is 8.6 days, and there are 12 stages that repeat at an 8.6-day interval[22],[23]. Inasmuch as intragastric administration of ethanol in mice mimicked alcoholism in man, MgIG appears to be a good candidate in the prevention of alcohol-induced testicular injuries.

Our data showed that there was greater protection against ethanol-induced injury in MH mice than ML mice. A previous study in rats demonstrated that MgIG was quickly distributed in organs and tissues after intravenous administration. At 5 min after administration, the concentration of MgIG in liver was significantly higher than that in testes. After 180 min, drug level in testes was similar to the liver[24]. Our data further indicates that MgIG is able to cross the blood-testis barrier and reach concentrations that can exert an antioxidant effect.

Overall, the results show that MgIG reduces the ethanol-induced testicular injury in mice. The protective effect may be accomplished by a decrease of oxidative stress and germ cell apoptosis. MgIG may provide a beneficial protective effect against the reproductive toxicity induced by exposure of ethanol.
